# Supplementary material for: IL-21 and IFNα therapy rescues terminally differentiated NK cells and limits SIV reservoir in ART-treated macaques
Source: Nat Commun. 2021 May 17;12:2866. doi: 10.1038/s41467-021-23189-7 (PMC8129202; doi:10.1038/s41467-021-23189-7)
Supplement: Supplementary file 1 — Supplementary Information [file 41467_2021_23189_MOESM1_ESM.pdf]

Supplementary Figure 1. Representative flow cytometry gating strategies.

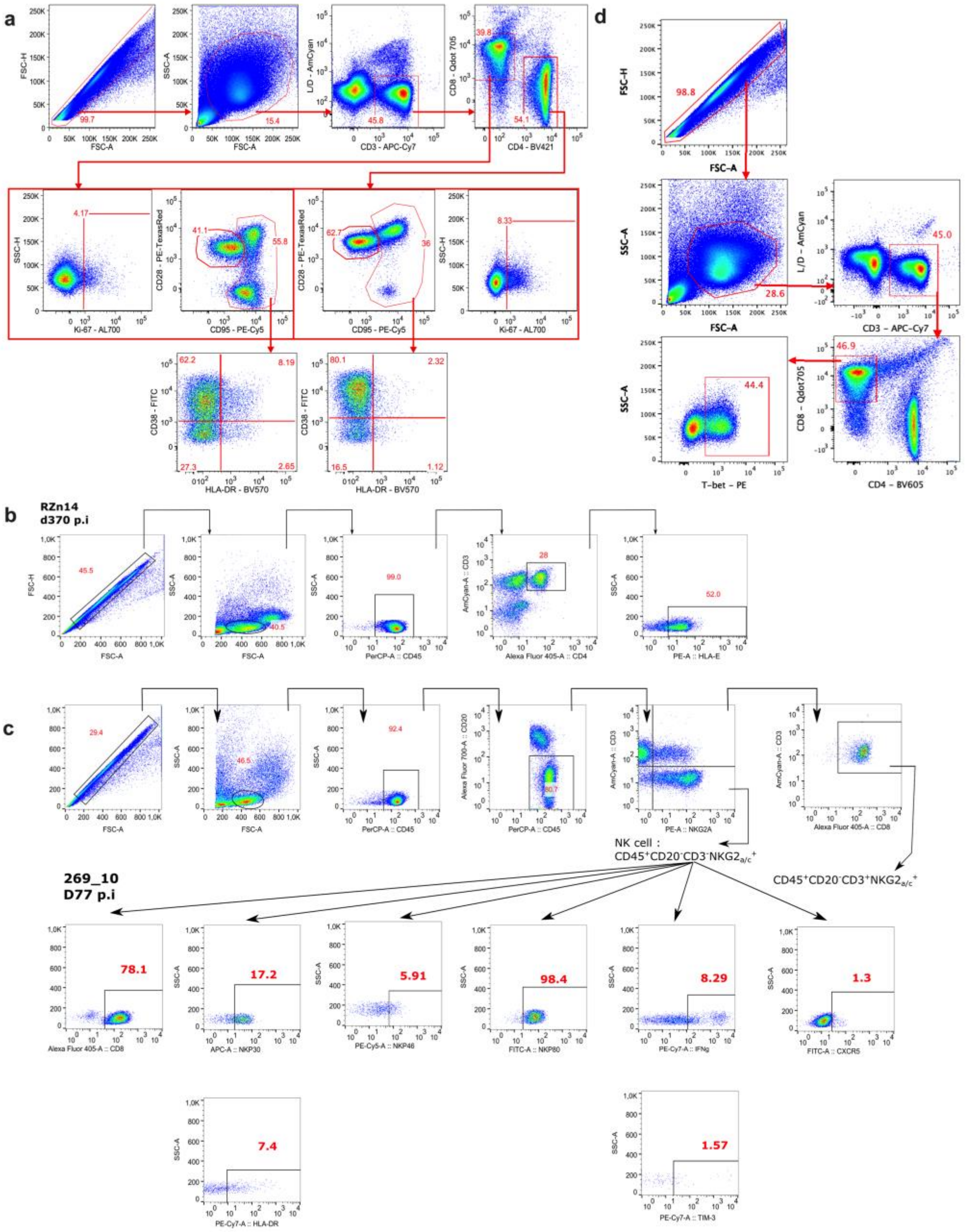

A representative flow cytometry gating strategy is shown for the immunophenotyping of (**a**,**d**) fresh and (**b**,**c**) cryo-preserved mononuclear cells from peripheral blood (PBMCs), lymph node (LN), and rectal biopsies (RB). Clusters of biomarkers within a single, common parental population are outlined with borders and the gating hierarchy is indicated by the directional arrows. Gate frequencies indicate the percentage of the population within the parental. Shown are PBMCs immunophenotyped for (**a**) T-cell activation and proliferation (RQm14 at d49 p.i.; 1 of 260 unique stains), (**b**) HLA-E expression in $CD4^+$ T-cells (RZn14 at d370 p.i.; 1 of 52 unique stains), (**c**) NK cell activation and homing (269_10 at d77 p.i.; 1 of 65 unique stains), and (**d**) T-bet expression in $CD8^+$ T-cells (RQm14 at d35 p.i., 1 of 91 unique stains), which were collected on an LSR II (BD Biosciences). Gating strategies corresponds to (**a**) Fig. 1d,e and Supplementary Fig. 2a-k; (**b**) Fig. 1g; (**c**) Fig. 1h; Fig. 3b,h; and Supplementary Fig. 7a-f; and (**d**) Supplementary Fig. 5a-c.

Supplementary Figure 2. IL-21 therapy following ART initiation transiently reduces T-cell immune activation and proliferation in rectal mucosa, but not in lymph nodes.

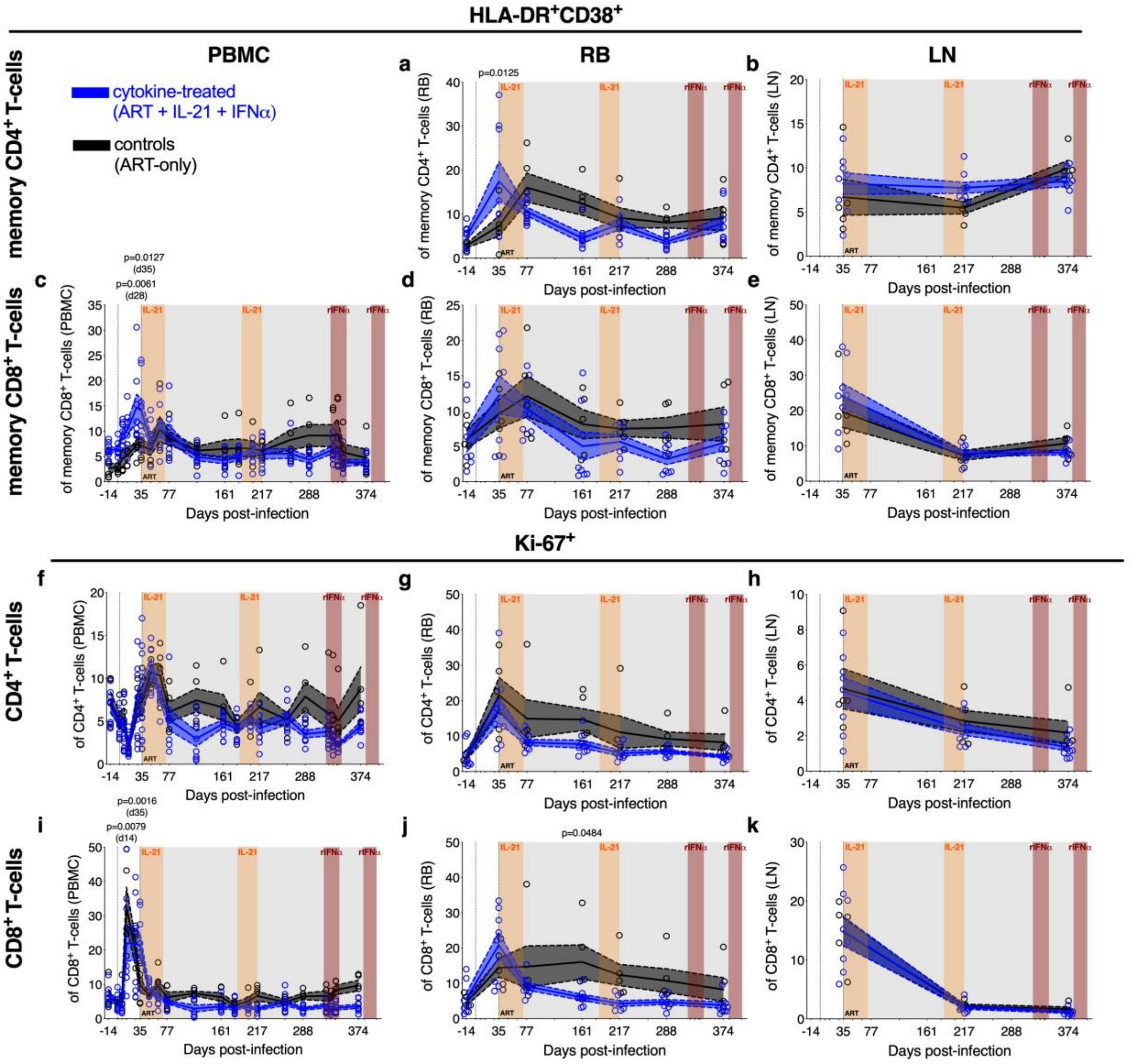

By flow cytometry the frequency of activation (HLA-DR⁺CD38⁺) was measured longitudinally within memory (CD95⁺) CD4⁺ T-cells from (**a**) RB and (**b**) LN, and within memory CD8⁺ T-cells from (**c**) PBMCs, (**d**) RB, and (**e**) LN. Likewise, the frequency of cellular proliferation (Ki-67⁺) was measured longitudinally within CD4⁺ T-cells from (**f**) PBMCs, (**g**) RB and (**h**) LN, and within CD8⁺ T-cells from (**i**) PBMCs, (**j**) RB, and (**k**) LN. A representative gating strategy is given in Supplementary Fig. 1a. (**a**,**b**,**c**,**d**,**e**,**f**,**g**,**h**,**i**,**j**,**k**) Data from individual RMs (staggered open circles) are overlaid against the mean (solid line) ± SEM (shaded region within the dashed lines): controls (ART-only, black; n=5) and cytokine-treated (ART + IL-21+ IFNα, blue; n=8). Treatment phases are indicated with the following background shading: IL-21 (orange), rIFNα (red), and ART (grey). Data were analyzed with two-sided (95% CI), two-way ANOVA with Bonferroni's correction for multiple comparisons with cross-sectional comparisons relative to controls.

Supplementary Figure 3. IL-21 therapy enhances the polyfunctionality of Th17/Th22 CD4^{+} cells in RB.

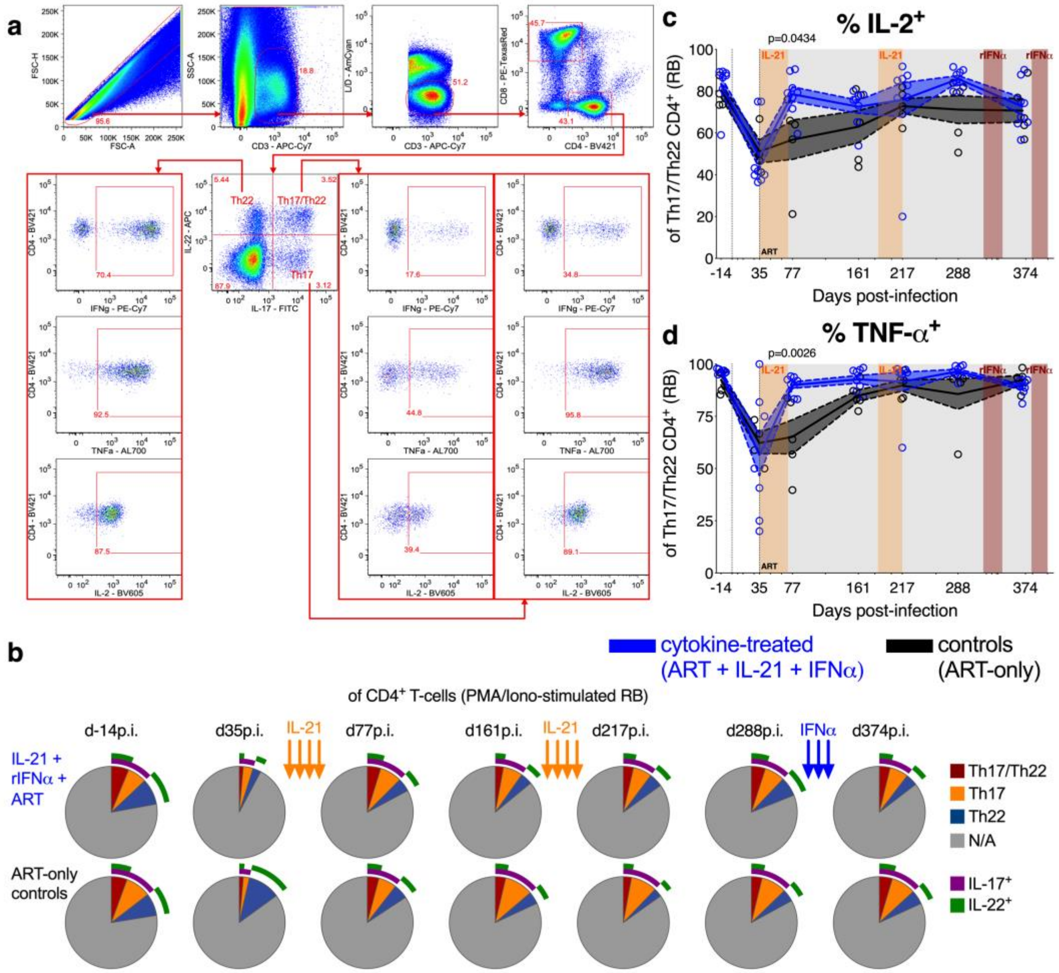

(**a**) A representative flow cytometry gating strategy is shown in pseudo color for the immunophenotyping of fresh rectal biopsy (RB) mononuclear cells following 4 hr *ex vivo* stimulation with PMA and ionomycin (269_10 at d-14 p.i., 1 of 91 unique stains). Clusters of biomarkers within a single, common parental population are outlined with borders in red and the gating hierarchy is indicated by the directional arrows in red. (**b**) Within stimulated RB memory CD4^{+} T-cells, the expression of intracellular IL-17 (purple) and IL-22 (green), as indicated by the outer, concentric rings, were used to determine the average distribution of Th17/Th22 (red), Th17 (orange), Th22 (blue) subsets of the total parental pool (i.e. those lacking IL-17 or IL-22 expression, N/A). The distribution of these subsets was analyzed over time and by treatment condition (cytokine-treated, n=8; controls, n=5) with a Permutation test in SPICE for which no comparisons were significant. Likewise, the frequency of (**c**) IL-2^{+} and (**d**) TNF-α^{+} cells were measured in stimulated RB Th17/Th22 CD4^{+} cells. (**c**,**d**) Treatment phases are indicated with the following background shading: IL-21 (orange), rIFNα (red), and ART (grey). Data from individual RMs (staggered, open circles) are overlaid against the mean (solid line) ± SEM (shaded region within the dashed lines): control (ART-only, black; n=5) and cytokine-treated (ART + IL-21+ IFNα, blue; n=8). Data were analyzed with a two-sided (95% CI), two-way ANOVA with Bonferroni's correction for multiple comparisons with cross-sectional comparisons relative to controls.

Supplementary Figure 4. Interferon-stimulated genes are upregulated during chronic SIV infection and fail to fully normalize despite suppressive ART.

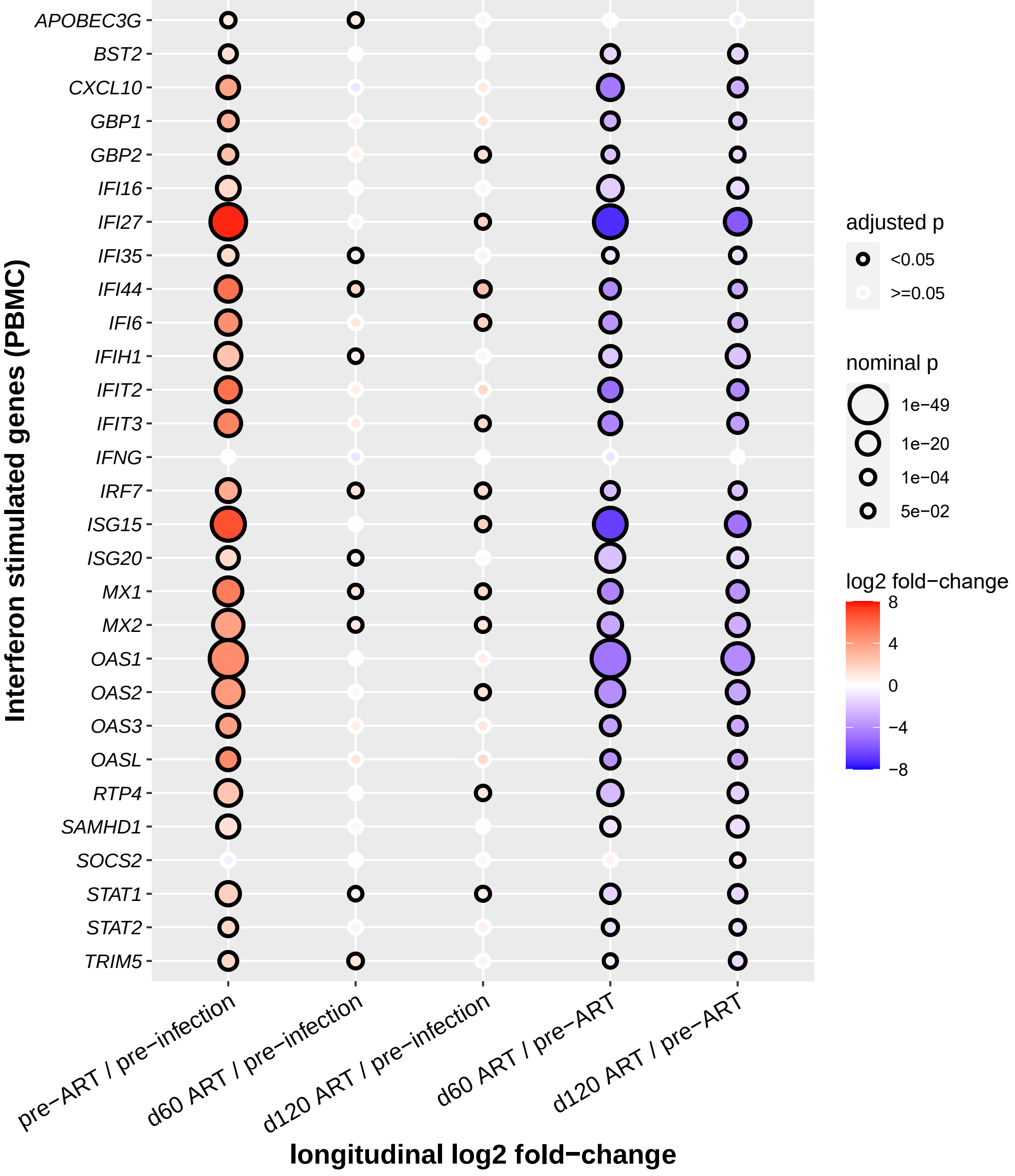

RMs (n=6) were infected intravenously with $SIVmac_{251}$ and treated with an ART regimen consisting of 3TC, tenofovir disoproxil fumarate (TDF), brecanavir, and cabotegravir. RNA-seq was performed on PBMCs and per each interferon (IFN)-stimulated gene (at left) the average log_2 fold-change in expression was calculated between the indicated time point (below) and those at pre-infection or pre-ART (chronic infection). The log_2 fold-change in gene expression is represented as a double-gradient heatmap and the size of each data point corresponds inversely to the log_{10}-transformed nominal p-value with significant ($p<0.05$) adjusted p-values represented by a black border. Using DESeq2, data were analyzed with a two-sided (95% CI) Wald test using the Benjamini-Hochberg method for multiple comparisons.

Supplementary Figure 5. Cytotoxic T Lymphocyte responses are not enhanced by IL-21 or rIFNα therapy amid ongoing ART.

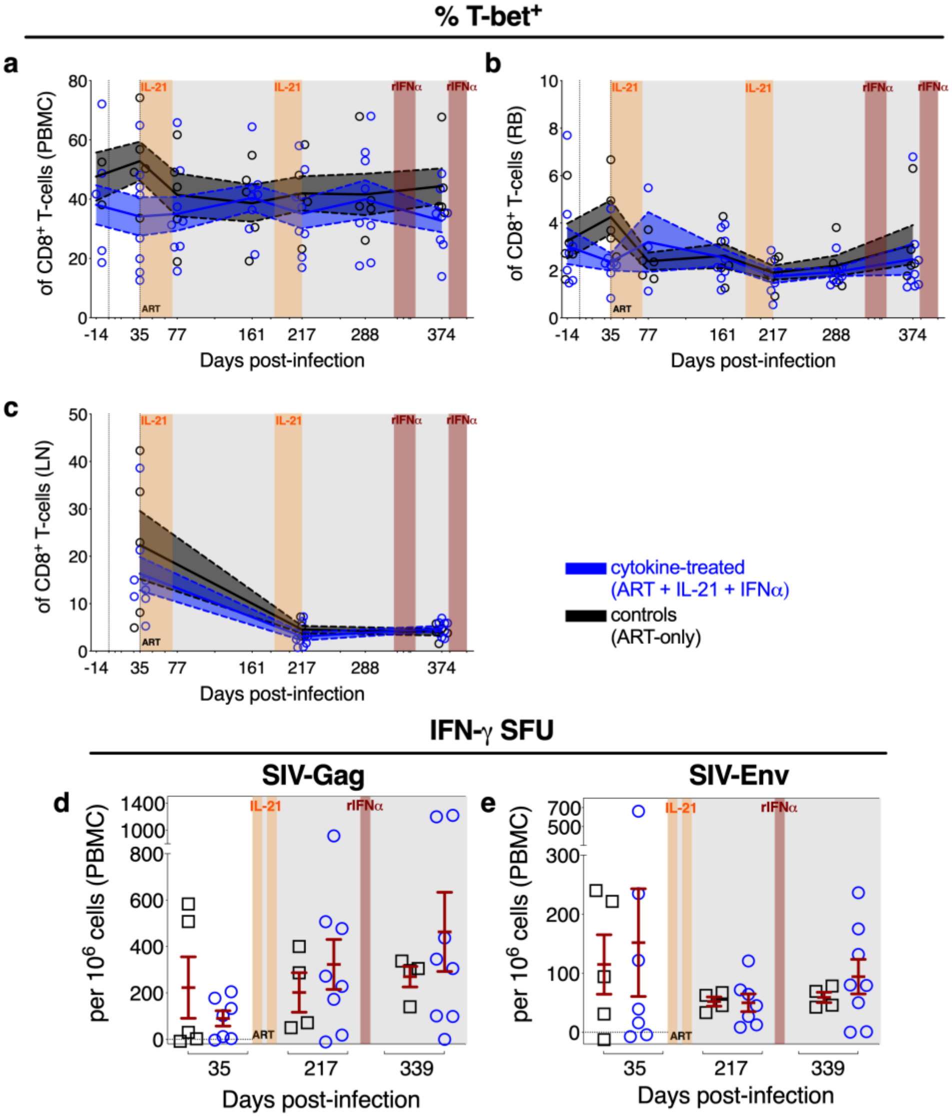

The frequency of T-bet$^+$ cells were measured by flow cytometry in CD8$^+$ T-cells from (**a**) PBMCs, (**b**) RB, and (**c**) LN. (**a**,**b**,**c**) Data from individual RMs (staggered open circles) are overlaid against the mean (solid line) ± SEM (shaded region within the dashed lines): control (ART-only, black; n=5) and cytokine-treated (ART + IL-21+ rIFNα, blue; n=8). A representative gating strategy is given in Supplementary Fig. 1d. The number of interferon gamma (IFN-γ) spot forming units (SFU) per 10^6 PBMCs were quantified by ELISpot following *ex vivo* stimulation with either (**d**) SIV-Gag or (**e**) SIV-Env peptide pools per each treatment condition: cytokine-treated (n=8) and controls (n=5). ELISpots were performed in triplicate; DMSO mock-stimulated controls were background subtracted; and RMs that failed to produce any SFU were excluded from analysis. (**d**,**e**) Data from individual RMs are overlaid against the mean ± SEM (in red). (**a**,**b**,**c**,**d**,**e**) Treatment phases are indicated with the following background shading: IL-21 (orange), rIFNα (red), and ART (grey). Data were analyzed with two-sided (95% CI), two-way ANOVA with Bonferroni's correction for multiple comparisons with cross-sectional comparisons relative to controls.

Supplementary Figure 6. NK cell CD107a expression and representative flow cytometry plots for the quantification of Env-specific activity.

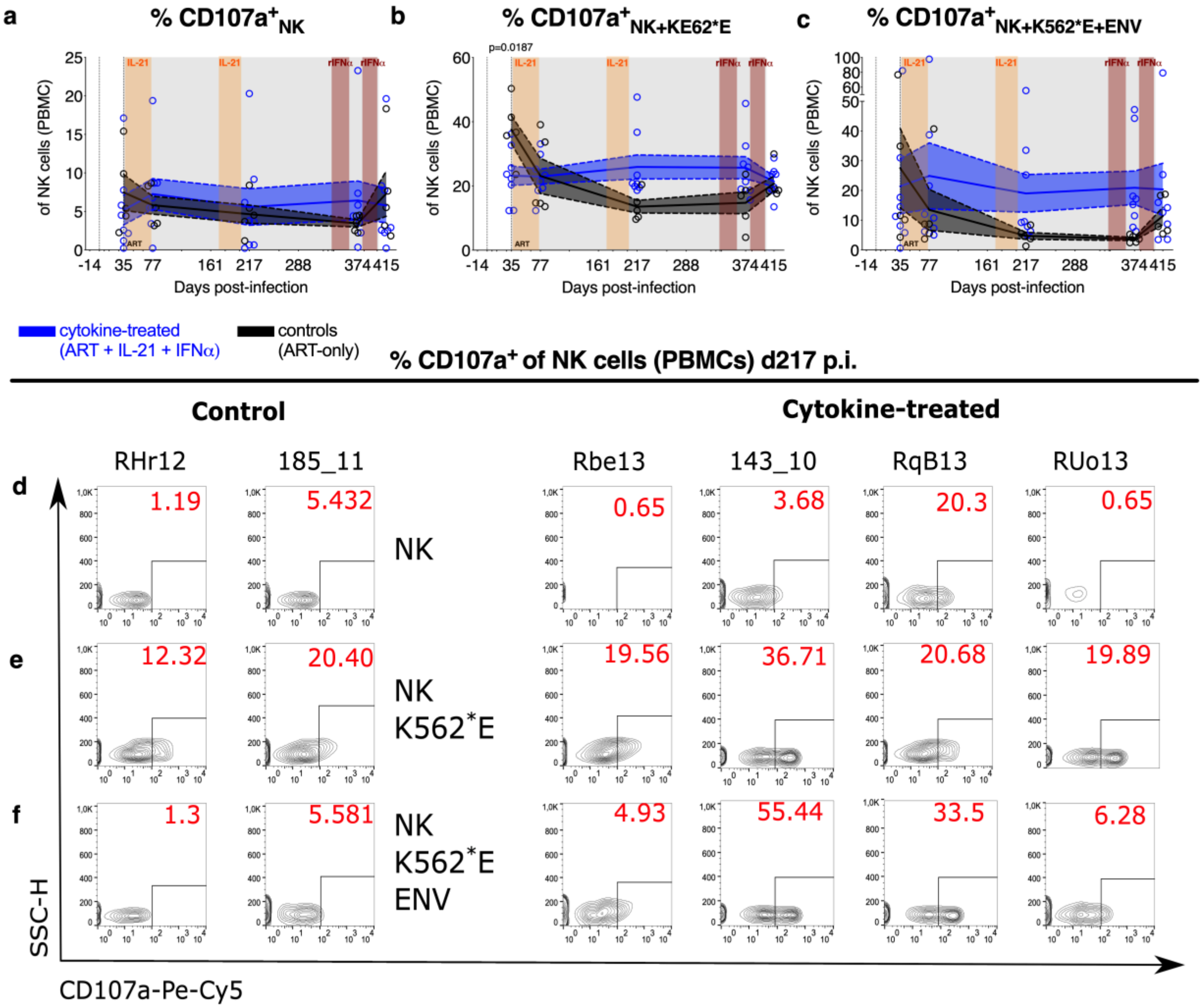

The frequency of CD107a+ NK cells was accessed by flow cytometry in the following culture conditions: (**a**) NK cells cultured alone (i.e. NK), (**b**) co-cultured with K562-E*0101 cells without peptide (i.e. NK+K562*E; d35 p=0.0187), or (**c**) co-cultured with K562-E*0101 cells loaded with $SIVmac_{239/251}$-Env peptides (i.e. NK+K562*E+ENV). (**a**,**b**,**c**) Data from individual RMs (staggered, open circles) are overlaid against the mean (solid line) ± SEM (shaded region within the dashed lines): control (ART-only, black; n=5) and cytokine-treated (ART + IL-21+ rIFNα, blue; n=8). Treatment phases are indicated with the following background shading: IL-21 (orange), rIFNα (red), and ART (grey). Data were analyzed with two-sided, two-way (95% CI) ANOVA with Bonferroni's correction for multiple comparisons with cross-sectional comparisons relative to controls. Representative flow cytometry contour plots with outliers are shown within NK cells ($CD45^+CD20^-CD3^-NKG2a/c^+$) for CD107a expression by SSC-H for each of the culture conditions: (**d**) NK, (**e**) NK+K562*E, or (**f**) NK+K562*E+ENV. Representative plots are shown at d217 p.i. for two controls (RHr12 and 185_11) and four cytokine-treated RMs (RBe13, 143_10, RQb13, and RUo13; 18 of 195 unique stains). The frequency of each gate of the parental population is given in red text and data was collected on an LSRFortessa (BD Biosciences). Representative stains also corresponds to Fig. 1i, Fig. 3i, and Fig. 5c,d.

Supplementary Figure 7. The expression of activating co-receptors and other signatures of maturation on NK cells were not regulated by IL-21 or rIFNα therapy amid ongoing ART.

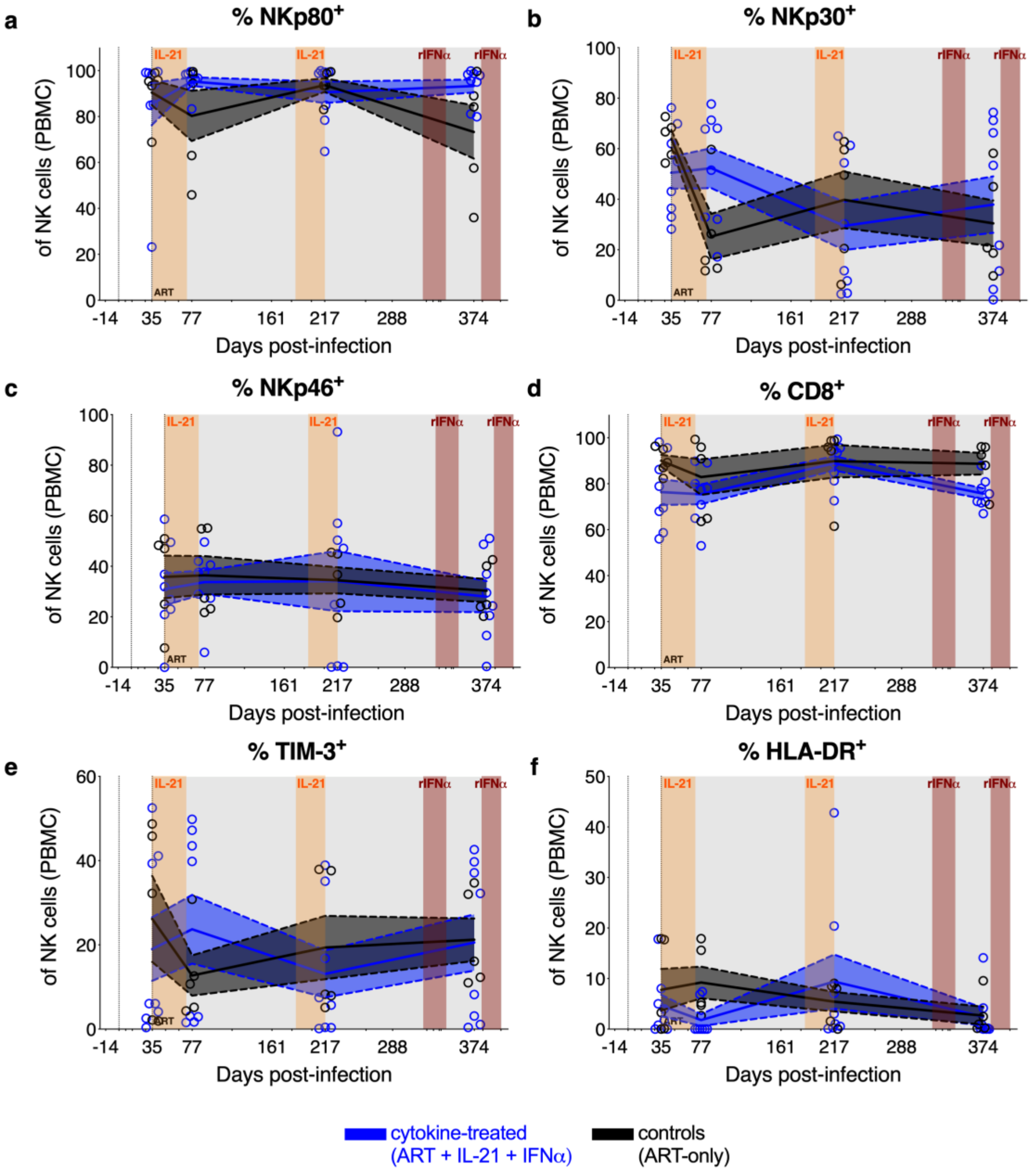

In PBMCs the frequency of NK cells ($CD45^{+}CD20^{-}CD3^{-}NKG2a/c^{+}$) expressing activating natural cytotoxicity receptors (**a**) NKp80, (**b**) NKp30, (**c**) NKp46 were quantified by flow cytometry as were other biomarkers of NK cell maturation: (**d**) CD8, (**e**) TIM-3, and (**f**) HLA-DR. (**a**,**b**,**c**,**d**,**e**,**f**) Data from individual RMs (staggered open circles) are overlaid against the mean (solid line) ± SEM (shaded region within the dashed lines): control (ART-only, black; n=5) and cytokine-treated (ART + IL-21+ IFNα, blue; n=8). Treatment phases are indicated with the following background shading: IL-21 (orange), rIFNα (red), and ART (grey). Data were analyzed with two-sided (95% CI), two-way (95% CI) ANOVA with Bonferroni’s correction for multiple comparisons with cross-sectional comparisons relative to controls. Corresponds to the gating strategy shown in Supplementary Fig. 1c.

Supplementary Figure 8. Representative flow cytometry gating strategy of NK cell immunophenotyping.

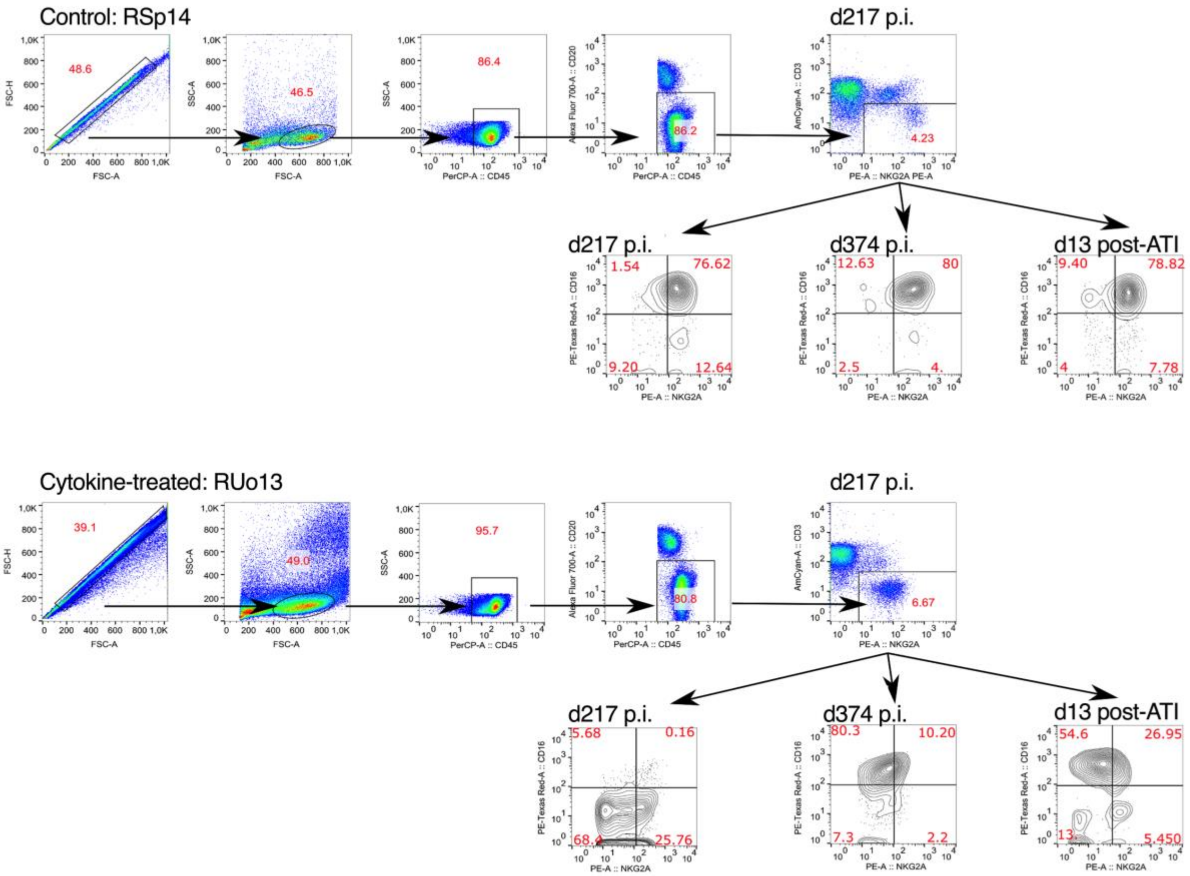

A representative flow cytometry gating strategy is shown in pseudo color for the immunophenotyping of cryo-preserved PBMCs from d217 p.i. per NK cell ($CD45^{+}CD20^{-}CD3^{-}$ $NKG2a/c^{+}$) characterization. The gating hierarchy is indicated by the directional arrows in black and the frequency of each gate of the parental population is given in red text. Shown are PBMCs (6 of 65 unique stains) from a control (RSp14) and cytokine-treated (RUo13) RM collected on an LSRFortessa (BD Biosciences). Representative contour plots with outliers are given within bulk NK cells for discrimination of the differentiation stages (NKG2a/c versus CD16) at d217 p.i., d374 p.i., and d13 post-ATI as indicated. Corresponds to Fig. 3a,c-f; Fig. 5b; Supplementary Fig. 9; and Supplementary Fig. 10f.

Supplementary Figure 9. Cytokine treatment redistributes, but does not expand, NK cell differentiation subsets with innate degranulation activity.

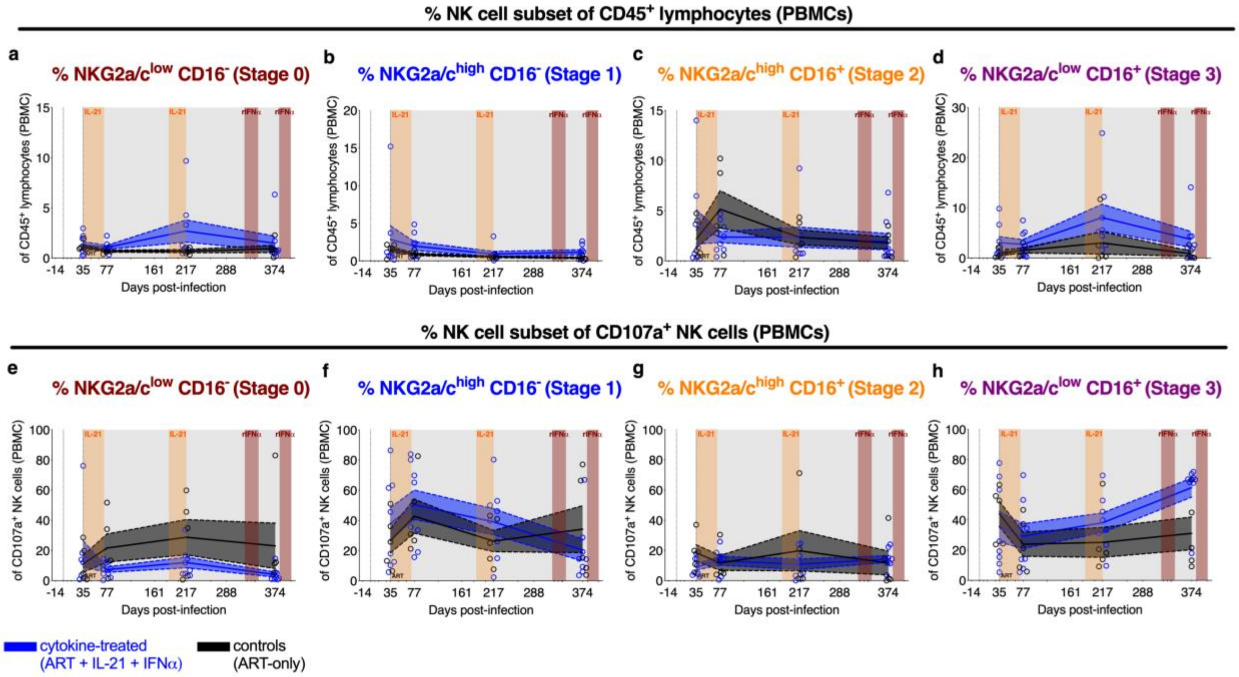

The distribution of NK cell (CD45$^+$CD20$^-$CD3$^-$NKG2a/c$^+$) differentiation stages were measured as a frequency of CD45$^+$ lymphocytes in cryo-preserved PBMCs by flow cytometry. The differentiation stages were defined as follows: (**a**) Stage 0 (red, NKG2a/c^{low}CD16$^-$), (**b**) Stage 1 (blue, NKG2a/c^{high}CD16$^-$), (**c**) Stage 2 (orange, NKG2a/c^{high}CD16$^+$), and (**d**) Stage 3 (purple, NKG2a/c^{low}CD16$^+$). *Ex vivo* innate NK cell activity (i.e. CD107a surface expression) was quantified by flow cytometry. Within CD107a$^+$ NK cells, the distribution of the NK cell differentiation stages was quantified as a frequency of parent: (**e**) Stage 0, (**f**) Stage 1, (**g**) Stage 2, and (**h**) Stage 3. (**a**,**b**,**c**,**d**,**e**,**f**,**g**,**h**) Data from individual RMs (staggered open circles) are overlaid against the mean (solid line) ± SEM (shaded region within the dashed lines): control (ART-only, black; n=5) and cytokine-treated (ART + IL-21+ IFNα, blue; n=8). Treatment phases are indicated with the following background shading: IL-21 (orange), rIFNα (red), and ART (grey). Data were analyzed with two-sided, two-way (95% CI) ANOVA with Bonferroni's correction for multiple comparisons with cross-sectional comparisons relative to controls. Corresponds to the gating strategy shown in Supplementary Fig. 8.

Supplementary Figure 10. Cytokine treatment alters early viral rebound kinetics following ATI while not impacting viral reservoir content.

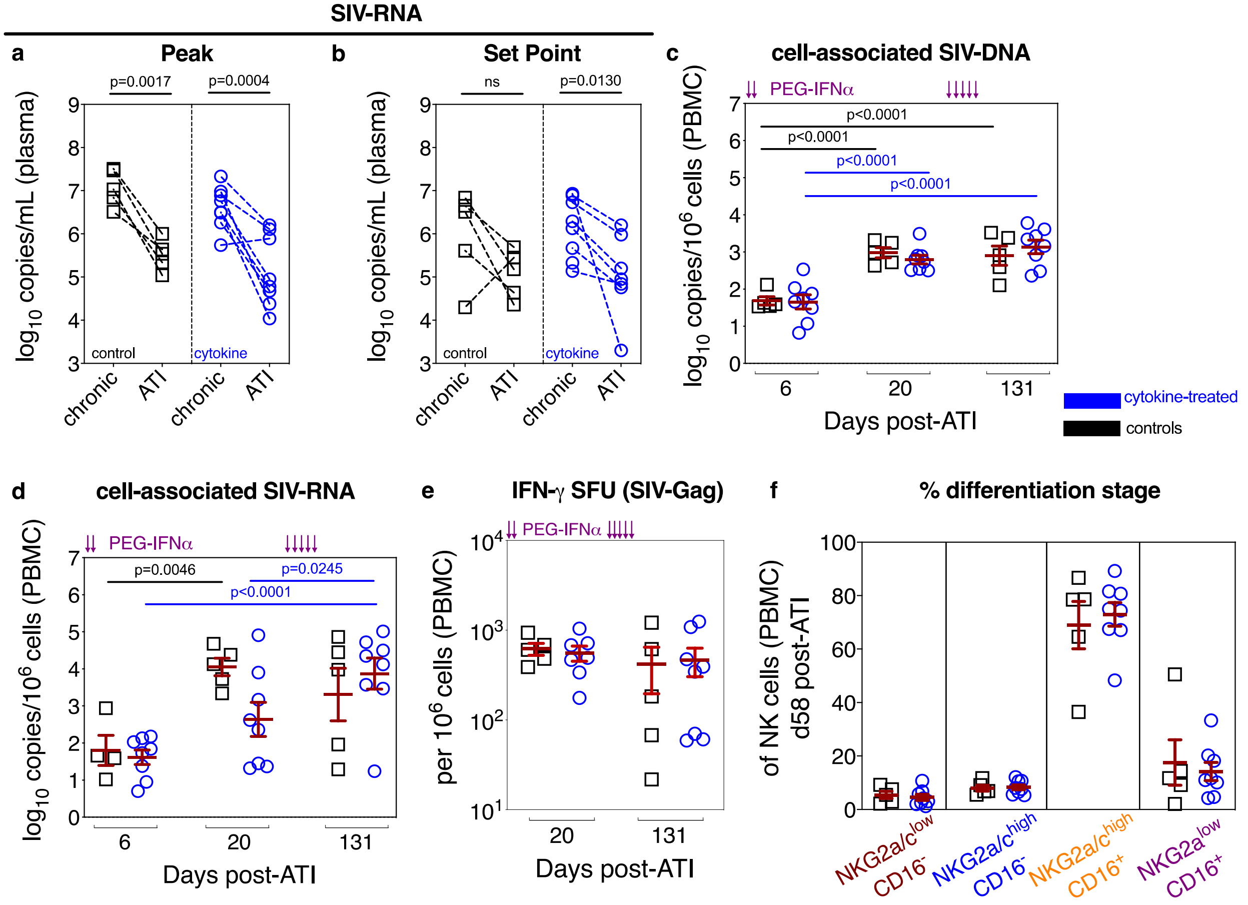

Plasma viral loads (SIV-RNA copies per mL) were measured by qRT-PCR following ART analytic treatment interruption (ATI). Individual plasma viral loads (log_{10} copies/mL) at (**a**) the initial peak and (**b**) the set point (d35 p.i. versus d51 ATI) were represented as before/after plots between chronic infection prior to ART initiation (chronic) and post-ATI (ATI): controls (prior ART-only, black; n=5 RMs) and cytokine-treated (prior ART + IL-21+ rIFNα with ongoing PEG-IFNα, blue; n=8 RMs). The cell-associated (**c**) SIV-DNA and (**d**) -RNA content (log_{10} copies per 10^6 PBMCs) were quantified by qRT-PCR following ATI: control (n=5 RM) and cytokine-treated (n=8 RM). (**e**) The number of IFN-γ spot forming units (SFU) per 10^6 PBMCs were quantified by ELISpot following *ex vivo* stimulation with SIV-Gag peptide pools: control (n=5) and cytokine-treated (n=8). (**f**) In PBMCs at d58 post-ATI, the distribution of the differentiation subsets was measured by flow cytometry as a frequency of NK cells for control (n=5) and cytokine-treated (n=8) RMs. NK cell maturation stages were defined as follows: Stage 0 (red, NKG2a/$c^{low}CD16^-$), Stage 1 (blue, NKG2a/$c^{high}CD16^-$), Stage 2 (orange, NKG2a/$c^{high}CD16^+$), and Stage 3 (purple, NKG2a/$c^{low}CD16^+$). (**c**,**d**,**e**,**f**) Data from individual RMs are given as color-coded, open shapes overlaid against the mean ± SEM (red). Data were analyzed with (**a**,**b**,**c**,**d**,**e**) a two-sided, two-way (95% CI) ANOVA with Bonferroni's correction for multiple comparisons (**a**,**b**,**c**,**d**,**e**) across treatments and time or (**f**) cross-sectionally relative to controls.

Supplementary Table 1. Rhesus macaque characteristics

Animal ID[a]	Sex	Age (months) at infection	SIV-RNA copies/mL (plasma) pre-ART[b]	#CD4 pre-ART[c]	*Mamu*-A*01 status	Treatment: ART-only controls	Treatment: ART + IL-21 + rIFNα + PEG-IFNα
219_03	F	147	4.48E+06	538	-	✓	
172_10 †	F	63	8.11E+05	740	-		✓
RHr12	F	87	4.11E+05	357	+	✓	
RYb12	F	100	2.01E+04	381	-	✓	
RPk11 Ω	F	113	4.80E+04	239	-	✓	
RNa12 Ω	F	101	3.59E+04	475	+	✓	
RQm14	F	53	7.79E+06	457	-		✓
143_10	F	65	1.36E+06	472	-		✓
RSp14	F	52	3.25E+06	184	+	✓	
185_11	F	50	6.86E+06	345	-	✓	
RQb13	F	78	4.69E+05	418	+		✓
269_10	F	64	5.39E+06	279	-		✓
RBe13	F	78	1.38E+05	641	-		✓
RZn14	F	53	8.46E+06	844	-		✓
ROi15	F	41	1.96E+06	371	-		✓
RUo13	F	76	2.13E+05	479	-		✓

[a]RM were excluded from analyses due to either reaching humane endpoints related to AIDS progression (†) or excluded to analyze post-treatment control (PTC; Ω).
[b]The number of SIV-RNA copies per mL of plasma were quantified by qRT-PCR in chronic infection immediately prior to ART initiation (d35 p.i.).
[c]The number of $CD4^+$ T-cells per μL of peripheral blood at the pre-ART collection were determined by complete blood counts (CBCs) and flow cytometry.

Supplementary Table 2. Primers for qRT-PCR assays.

Assay[a]	Primer[b]	Primer name	Sequence[c]
Quantification cell-associated SIV-DNA and -RNA	forward primer	sGAG21	5′-GTCTGCGTCAT(dP)TGGTGCATTC-3′
	reverse primer	sGAG22	5′-CACTAG(dK)TGTCTCTGCACTAT(dP)TGTTTTG-3′
	probe	psGAG23	5′-FAM-CTTC(dP)TCAGT(dK)TGTTTCACTTTCTCTTCTGCG-BHQ1-3′.
	nested primer	SIVnestF01	5′-GATTTGGATTAGCAGAAAGCCTGTTG-3′
	nested primer	SIVnestR01	5′-GTTGGTCTACTTGTTTTTGGCATAGTTTC-3′
Determination of plasma viremia	forward primer	sGAG21	5′-GTCTGCGTCAT(dP)TGGTGCATTC-3′
	reverse primer	sGAG22	5′-CACTAG(dK)TGTCTCTGCACTAT(dP)TGTTTTG-3′
	probe	psGAG23	5′-FAM-CTTC(dP)TCAGT(dK)TGTTTCACTTTCTCTTCTGCG-BHQ1-3′

[a]Indicates which primers were utilized for each qRT-PCR assay listed in the Methods.
[b]Primers are directed against a highly conserved domain of $SIVmac_{239}$ *gag*.
[c]Probes are conjugated with BHQ1 (black hole quencher-1) and FAM (6-carboxyfluorescein). The forward primers, reverse primers, and probes contain neutral bases (dK and dP) at the indicated positions to avoid biasing in measuring non-$SIVmac_{239}$ viral sequences.
