# Supplementary material for: IL-21 and IFNα therapy rescues terminally differentiated NK cells and limits SIV reservoir in ART-treated macaques
Source: Nat Commun. 2021 May 17;12:2866. doi: 10.1038/s41467-021-23189-7 (PMC8129202; doi:10.1038/s41467-021-23189-7)
Supplement: Supplementary file 3 — Reporting summary [file 41467_2021_23189_MOESM3_ESM.pdf]

nature research

Corresponding author(s): Mirko Paiardini

Last updated by author(s): Dec 17, 2020

Reporting Summary

Nature Research wishes to improve the reproducibility of the work that we publish. This form provides structure for consistency and transparency in reporting. For further information on Nature Research policies, see our Editorial Policies and the Editorial Policy Checklist.

Statistics

For all statistical analyses, confirm that the following items are present in the figure legend, table legend, main text, or Methods section.

n/a	Confirmed	
☐	☒	The exact sample size (n) for each experimental group/condition, given as a discrete number and unit of measurement
☐	☒	A statement on whether measurements were taken from distinct samples or whether the same sample was measured repeatedly
☐	☒	The statistical test(s) used AND whether they are one- or two-sided<br>*Only common tests should be described solely by name; describe more complex techniques in the Methods section.*
☐	☒	A description of all covariates tested
☐	☒	A description of any assumptions or corrections, such as tests of normality and adjustment for multiple comparisons
☐	☒	A full description of the statistical parameters including central tendency (e.g. means) or other basic estimates (e.g. regression coefficient) AND variation (e.g. standard deviation) or associated estimates of uncertainty (e.g. confidence intervals)
☐	☒	For null hypothesis testing, the test statistic (e.g. F, t, r) with confidence intervals, effect sizes, degrees of freedom and P value noted<br>*Give P values as exact values whenever suitable.*
☒	☐	For Bayesian analysis, information on the choice of priors and Markov chain Monte Carlo settings
☒	☐	For hierarchical and complex designs, identification of the appropriate level for tests and full reporting of outcomes
☐	☒	Estimates of effect sizes (e.g. Cohen's d, Pearson's r), indicating how they were calculated

Our web collection on statistics for biologists contains articles on many of the points above.

Software and code

Policy information about availability of computer code

Data collection	Flow Cytometry data was collected using FACS DiVa software on an LSR II or LSR Fortessa (BD Biosciences). RNA-seq data was collected on an Illumina HiSeq 3000 system. Plasma viremia and cell-associated SIV-RNA and -DNA were collected using a hybrid "real time/digital" format qRT-PCR platform (Biorad).
Data analysis	Flow Cytometry data was processed using FlowJo version 9.9.6 per T-cells or version 10.4.2 per NK cells (TreeStar). Statistics were performed using GraphPad Prism version 8.1.2. RNA-seq data were mapped with RhesusGenome using STAR 2.5.2b with default alignment parameters; the abundance of transcript counts was performed with STAR using the HTSeq-count algorithm; and DESeq2 version 1.22.1 R package was used to produce normalized read counts and compute the differential expression estimation using the Wald test. Values of plasma viremia and cell-associated SIV-RNA and -DNA were calculated by interpolating individual cycle threshold (Ct) values onto a standard curve or using Poisson methods if not all reactions were positive. For the quantitative viral outgrowth assay, the frequency of cells producing replication competent virus were determined by the maximum-likelihood method utilizing IUPMStats and were expressed in infectious units per million (IUPM).

For manuscripts utilizing custom algorithms or software that are central to the research but not yet described in published literature, software must be made available to editors and reviewers. We strongly encourage code deposition in a community repository (e.g. GitHub). See the Nature Research guidelines for submitting code & software for further information.

Data

Policy information about availability of data

All manuscripts must include a data availability statement. This statement should provide the following information, where applicable:

- Accession codes, unique identifiers, or web links for publicly available datasets
- A list of figures that have associated raw data
- A description of any restrictions on data availability

~~Source data are provided for the performed correlations, as are the statistical readouts for the correlations and RNA-seq analyses. RNA-seq data are available in GenBank (uploaded with accession numbers pending). Other data, including custom code, that support the findings of this work are available from the corresponding author on reasonable request.~~

Field-specific reporting

Please select the one below that is the best fit for your research. If you are not sure, read the appropriate sections before making your selection.

[x] Life sciences [ ] Behavioural & social sciences [ ] Ecological, evolutionary & environmental sciences

For a reference copy of the document with all sections, see nature.com/documents/nr-reporting-summary-flat.pdf

Life sciences study design

All studies must disclose on these points even when the disclosure is negative.

Sample size	16 female Indian rhesus macaques (RMs; Macaca mulatta), all housed at the Yerkes National Primate Research Center (YNPRC) at Atlanta, Ga, were included in this study. Animals were between 41 and 147 months old at time of infection (Supplementary Table 1). 9 RMs were cytokine-treated; however, 172_10 was euthanized at day 66 p.i. due to rapid progression to AIDS-defining endpoints related to weight loss (cytokine-treated, n=8). ~~Of the 7 control RMs, 2 were defined as pre-treatment controllers (RPk11 and RNa12) and were excluded from analyses (controls, n=5).~~<br><br>~~Group sizes were selected based as extrapolated from previous studies demonstrating they provide approximately 80% statistical power to detect a mean difference of 1.5 standard deviations of greater for all analyses while not overdrawing animals from the colony.~~
Data exclusions	Select flow cytometry data were excluded based on the presence of aberrant staining or due to insufficient stopping gate events (i.e. live CD3 + T-cells; <120,000 events for LN/PBMC or <50,000 for RB. IFNg ELISpot data were excluded if no positive wells were detected. Prior to ART initiation, ~~the animals RPk11 and RNa12 mimicked virologic and immunologic features of controllers and were excluded from analyses.~~ QVOA was performed on 4 control and 6 cytokine-treated RMs based on cellular yields.
Replication	No attempts were made at replication due to the duration of the in vivo study (2 years) and due to substantial costs associated with animal acquisition, per diem charges, and surgical costs. Certain assays were performed as ~~replicates~~: plasma viremia (6x), cell-associated SIV-RNA/DNA (~~12~~X), IFNg ELISpot (3X), and QVOA (3x replicates in 3 serial dilutions).
Randomization	~~Animals were treatment stratified to balance for set point plasma viremia prior to ART initiation (Supplementary Table 1).~~
Blinding	Blinding of primary investigators was not possible as they were responsible for the distribution of compounds for administration and treatment stratification. Secondary investigators were blinded to treatment group and experimental design prior to the performed analyses, including all of those performed per NK cell characterization.

Reporting for specific materials, systems and methods

We require information from authors about some types of materials, experimental systems and methods used in many studies. Here, indicate whether each material, system or method listed is relevant to your study. If you are not sure if a list item applies to your research, read the appropriate section before selecting a response.

Materials & experimental systems

n/a	Involved in the study	
[ ]	[x]	Antibodies
[ ]	[x]	Eukaryotic cell lines
[x]	[ ]	Palaeontology and archaeology
[ ]	[x]	Animals and other organisms
[x]	[ ]	Human research participants
[x]	[ ]	Clinical data
[x]	[ ]	Dual use research of concern

Methods

n/a	Involved in the study	
[x]	[ ]	ChIP-seq
[ ]	[x]	Flow cytometry
[x]	[ ]	MRI-based neuroimaging

Antibodies

Antibodies used

The following antibodies were utilized for the longitudinal staining panel per T-cell and B-cell characterization: anti-CD21-PE (clone B-ly4, 10 uL, cat. 555422), anti-CD28-PE-CF594 (clone CD28.2, cat. 562296), anti-CD95-PE-Cy5 (clone DX2, 10 uL, cat. 559773), anti-CCR7-PE-Cy7 (clone 3D12, 7.5 uL, cat. 557648), anti-CD45RA-APC (clone 5H9, 10 uL, cat. 561210), anti-Ki-67-AL700 (clone B56, cat. 561277), and anti-CD3-APC-Cy7 (clone SP34-2, cat. 557757) all from BD Biosciences; anti-HLA-DR-BV570 (clone L243, cat. 307638), anti-CD27-BV605 (clone O323, 10 uL, cat. 302830), anti-CD20-PerCP-Cy5.5 (clone 2H7, cat. 302326), and anti-CD4-BV421 (clone OKT4, 2.5 uL, cat. 317438) all from Biolegend; anti-CD38-FITC (clone AT-1, cat. 60131FI) from STEMCELL Technologies; and anti-CD8-Qdot705 (clone 3B5, 1 uL, cat. Q10059) and Live/Dead Fixable Aqua (AmCyan, 2 uL of 1:20 PBS dilution, cat. L34957) from Thermo Fisher Scientific (Supplementary Fig. 1). mAbs were added at the manufacturer's recommended test volume per 10^6 mononuclear cells (1:100 dilution) unless otherwise noted, and cells were fixed and permeabilized with a Cytofix/Cytoperm kit (BD Biosciences).

Cryo-preserved PBMCs were stained as previously described to characterize NK cell subsets using a panel of the following mAbs: anti-CD3-PacBlue (clone SP34-2, 5 uL, cat. 558124), anti-CD45-PerCP (clone D058-1283, 5 uL, cat. 558411), and anti-CD69-APC-Cy7 (clone FN50, 5 uL, cat 557756) all from BD Biosciences; anti-CD8-VioBlue (clone BW135/80, 2.5 uL, cat. 130-094-152), anti-NKp80-FITC (clone 4A4.D10, 8 uL, cat. 130-094-843), anti-NKp30-APC (clone AF29-4D12, 8 uL, cat. 130-092-484) all from Miltenyi Biotec; and anti-CD20-AL700 (clone 2H7, 8 uL, cat. 56-0209) from eBioscience; anti-TIM-3-PE-Cy7 (clone F38-2E2, 5 uL, cat. 345014); and anti-NKG2a-PE (clone Z199, 8 uL, cat. IM3291U), anti-CD16-ECD (clone 3G8, 5 uL, cat. 41116015), and anti-NKp46 (clone BAB281, 8 uL, cat. A66902) all from Beckman Coulter. A secondary immunophenotyping panel consisted of the following mAbs: anti-CD3-V500 (clone SP34-2, 5 uL, cat. 560770), anti-CD45-PerCP (clone D058-1283, 5 uL, cat. 558411), anti-CD95-APC (clone DX2, 5 uL, cat. 558814), anti-CD107a-PE-Cy5 (clone HA43, 5 uL, cat. 555802), and anti-CCR7-BV711 (clone 3D12, 5 uL, cat. 563712) all from BD Biosciences; anti-CD8-VioBlue (clone BW135/80, 2.5 uL, cat. 130-094-152) from Miltenyi Biotec; anti-CD16-ECD (clone 3G8, 5 uL, cat. 41116015) and anti-NKG2a-PE (clone Z199, 8 uL, cat. IM3291U) from Beckman Coulter; anti-HLA-DR-PE-Cy7 (clone L243, 5 uL, cat. 307616) and anti-PD-1-APC-Cy7 (clone EH12.2H7, 5 uL, cat. 329922) from Biolegend; and anti-CXCR5-FITC (clone MU5UBEE, 5 uL, cat. 15566616) from eBioscience. A variant of this panel used anti-HLA-E-PE (clone 3D12HLA-E, 6 uL, cat. NBP2-00277) from R&D Systems. The anti-NKG2a mAb recognizes both NKG2a and NKG2c in macaques.

Fresh mononuclear cells derived from RB punches were stained with the following panel to quantify cytokine expression: anti-CD8-PE-CF594 (clone RPA-T8, clone 562282), anti-CD95-PE-Cy5 (clone DX2, 10 uL, cat. 559773), anti-IFNg-PE-Cy7 (clone B27, cat. 557643), anti-TNFa-AL700 (clone Mab11, 1 uL, cat. 557996), and anti-CD3-Cy7APC (clone SP34-2, cat. 557757) all from BD Biosciences; anti-CD4-BV421 (clone OKT4, 4 uL, cat. 317434) and anti-IL-2-BV605 (clone MQ1-17H12, 1 uL, cat. 500332) both from Biolegend; and anti-IL-17-FITC (clone eBio64DEC17, cat. 53-7179-42), anti-IL-22-APC (clone IL22JOP, 5 uL, cat. 17-7222-82), and Live/Dead Fixable Aqua (AmCyan, 2 uL of 1:20 PBS dilution, cat. L34957) from Thermo Fisher Scientific. mAbs were added at the manufacturer's recommended test volume per 10^6 mononuclear cells (1:100 dilution) unless otherwise noted, and cells were fixed and permeabilized with a Cytofix/Cytoperm kit (BD Biosciences). The intracellular stain was incubated for 1 hr at room temperature in the dark.

To quantify NK cell activity the co-culture was performed in the presence of anti-CD107a-PE-Cy5 (clone HA43, 5 uL, cat. 555802; BD Biosciences) with BD GolgiStop (BD Biosciences) added 1 hr following initiation. Cells were then washed with PBS and stained with the following mAbs: anti-CD3-PacBlue (clone SP34-2, 5uL, cat. 558124), anti-CD20-PE-Cy7 (clone 2H7, 5 uL, cat. 560735), anti-CD69-APC-Cy7 (clone FN50, 5 uL, cat 557756), anti-CD45-PerCP (clone D058-1283, 5 uL, cat. 558411), and anti-IFN-g-AL700 (clone GB11, 8 uL, cat. 560213) all from BD Biosciences; anti-CD8-VioBlue (clone BW135/80, 2.5 uL, cat. 130-094-152) from Miltenyi Biotec; anti-CD16-ECD (clone 3G8, 5 uL, cat. 41116015), anti-NKG2a-PE (clone Z199, 8 uL, cat. IM3291U) all from Beckman Coulter; and anti-Perforin-FITC (clone PF-344, 5 uL, cat. 3465-7) from Mabtech. Cells were fixed and permeabilized with a Cytofix/Cytoperm kit (BD Biosciences) and intracellular stain was incubated a 4C for 15 min.

Validation

All mAbs were validated as being human reactive by the manufacturer with some being validated as rhesus reactive as indicated by databases maintained by the NHP Reagent Resource (MassBiologics). We, and others, have also previously demonstrated these mAb clones to be rhesus reactive:

Micci, L., et al. Interleukin-21 combined with ART reduces inflammation and viral reservoir in SIV-infected macaques. J Clin Invest 125, 4497-4513 (2015).

McGary, C.S., et al. CTLA-4(+)PD-1(-) Memory CD4(+) T Cells Critically Contribute to Viral Persistence in Antiretroviral Therapy-Suppressed, SIV-Infected Rhesus Macaques. Immunity 47, 776-788 e775 (2017).

Harper, J., et al. CTLA-4 and PD-1 dual blockade induces SIV reactivation without control of rebound after antiretroviral therapy interruption. Nat Med 26, 519-528 (2020).

Huot, N., et al. Natural killer cells migrate into and control simian immunodeficiency virus replication in lymph node follicles in African green monkeys. Nat Med 23, 1277-1286 (2017).

Bryceson, Y.T., et al. Functional analysis of human NK cells by flow cytometry. Methods Mol Biol 612, 335-352 (2010).

Eukaryotic cell lines

Policy information about cell lines

Cell line source(s)	The K-562 cell line was purchased from ATCC and transduced with a lentivirus expression vector for human HLA-E was purchases from Applied Biological Materials Inc. The 174xCEM cell line was supplied by Dr. Peter Cresswell via the NIH AIDS Reagent Program.
Authentication	None of the cell lines were authenticated.
Mycoplasma contamination	Cell lines were not tested for Mycoplasma contamination.
Commonly misidentified lines (See ICLAC register)	N/A

Animals and other organisms

Policy information about studies involving animals; ARRIVE guidelines recommended for reporting animal research

Laboratory animals	16 female Indian rhesus macaques (RMs; Macaca mulatta), all housed at the Yerkes National Primate Research Center (YNPRC) at Atlanta, Ga, were included in this study. Animals were between 41 and 147 months old at time of infection (Supplementary Table 1). 172_10 was euthanized at day 66 p.i. due to rapid progression to AIDS-defining endpoints related to weight loss. 5 RMs were utilized as ART-only controls and 2 RMs (RPk11 and RNa12) were projected to be controllers and were reassigned.<br><br>Group sizes were selected based as extrapolated from previous studies demonstrating they provide approximately 80% statistical power to detect a mean difference of 1.5 standard deviations of greater for all analyses while not overdrawing animals from the colony.
Wild animals	Study did not involve wild animals.
Field-collected samples	Study did not involve field-collected samples
Ethics oversight	All procedures were performed in accordance with institutional regulations and were approved by Emory University's Institutional Animal Care and Use Committee (IACUC; permit 3000434).

Note that full information on the approval of the study protocol must also be provided in the manuscript.

Flow Cytometry

Plots

Confirm that:

- [x] The axis labels state the marker and fluorochrome used (e.g. CD4-FITC).
- [x] The axis scales are clearly visible. Include numbers along axes only for bottom left plot of group (a 'group' is an analysis of identical markers).
- [x] All plots are contour plots with outliers or pseudocolor plots.
- [x] A numerical value for number of cells or percentage (with statistics) is provided.

Methodology

Sample preparation	All tissues were taken from SIVmac239-infected rhesus macaques as detailed in the study design (Fig. 1a). Tissues were collected and processed as previously described in McGary et al., Immunity, 2017. The RNA-seq data shown in Supplementary Fig. 4 was drawn from an external, historical cohort Dunham, R.M., et al. Pharmacologic inhibition of IDO blunts features of SIV-related chronic inflammation. in Conference on Retroviruses and Opportunistic Infections (Seattle, WA, 2017).<br><br>The immunophenotyping of T-cells were performed on fresh tissue and NK cells were characterized from cryo-preserved PBMCs. Cells were fixed and permeabilized with a Cytofix/Cytoperm kit (BD Biosciences).<br><br>NK cell cytolytic activity was determined through expression of cell surface CD107a. Cryo-preserved PBMCs were labelled with anti-NKG2a/c-PE (clone Z199, cat. IM3291U; Beckman Coulter) conjugated with anti-PE MicroBeads (Miltenyi Biotec), magnetically isolated according to the manufacturer's instructions, and cells were incubated overnight at 37C with 5% CO2 in RPMI supplemented with 10% (v/v) FBS, 100 U/mL IL-2 and 10 ng/mL of IL-15. For 6 to 8 hr, cells were incubated in a 1:5 ratio with MHC-class-I-devoid target cell line K-562. The co-culture was performed in the presence of anti-CD107a-PE-Cy5 (clone HA43, 5 uL, cat. 555802; BD Biosciences) with BD GolgiStop (BD Biosciences) added 1 hr following initiation. Cells were then washed with PBS and stained. Cells were fixed and permeabilized with a Cytofix/Cytoperm kit (BD Biosciences) and intracellular stain was incubated a 4C for 15 min.<br><br>Th17 and Th22 cells were defined as the frequency IL-17 and IL-22 producing CD4+ T-cells upon ex vivo stimulation with PMA and ionomycin. Fresh mononuclear cells derived from RB punches were incubated for 4 hr at 37°C in RPMI 1640 medium

	supplemented with 10% fetal bovine serum containing PMA (80 ng/mL), calcium ionophore A23187 (500 ng/mL), brefeldin A (10 ug/mL), and BD GolgiStop with monensin (7x10^-4 dilution). Cells were fixed and permeabilized with a Cytofix/Cytoperm kit (BD Biosciences). The intracellular stain was incubated for 1 hr at room temperature in the dark.
Instrument	LSR II (BD Biosciences) per T-cell panels and LSRFortessa (BD Biosciences) per NK cell panels.
Software	Cytometers were driven by FACS DiVa software (BD Biosciences). Analyses performed with FlowJo version 9.9.6 or 10.4.2 (TreeStar).
Cell population abundance	FASC sorting of cellular subsets was not performed.<br><br>Immunophenotyping acquisition was performed on a minimum of 120,000 live CD3+ T-cells for PBMC and LN, and a minimum of 50,000 live CD3+ T-cells in RB, when possible.
Gating strategy	Mononuclear cells were defined as laying on the diagonal of FSC-H versus FSC-A, and lymphocytes were gated from SSC-A versus FSC-A.<br><br>Per T-cell characterization, CD4+ and CD8+ T-cells were pre-gated as live CD3+ lymphocytes, and memory subsets were gated as CD95+CD28+/- (Supplementary Fig. 1 and Supplementary Fig. 3a).<br><br>Per NK cell characterization, singlet lymphocytes were gated as CD45+, CD20-, and CD3- NKG2a/c+ (Supplementary Fig. 8). NK cell differentiation subsets were defined based on CD16 expression within the NKG2a/c_low and NKG2a/c_high populations.

☒ Tick this box to confirm that a figure exemplifying the gating strategy is provided in the Supplementary Information.
